# Types of Mankind: Or Ethnological Researches, Based upon the Ancient Monuments, Paintings, Sculptures, and Crania of Races, and upon Their Natural, Geographical, Philosophical, and Biblical History

**Published:** 1854-06

**Authors:** 


ART. IV. — *Types of Mankind: or Ethnological Researches, based upon the ancient monuments, paintings, sculptures, and crania of races, and upon their natural, geographical, philological, and biblical history; Illustrated by selections from the unedited papers of Samuel George Morton, M. D., (late President of the Academy of Natural Sciences at Philadelphia,) and by additional contributions from Prof. L. Agassiz, L. L. D.; W. Usher, M. D.; and Prof. H. S. Patterson, M. D.;* By J. C. Nott, M. D., and Geo. R. Gliddon. Philadelphia: Lippincott, Grambo & Co., 1854.

Our necessities of time compelled us to defer to the present number our notice of this work. It is a duty which we approach with reluctance. The questions involved reach beyond mere scientific interest, and attack much that we are accustomed to regard as unimpeachable truth. The distinguished names upon its title page demand for it a calm investigation, which we fear it may not receive from all quarters; while the dignified and manly tone which it manifests, no less than the deep interest which accompanies its perusal, recommend it to the careful and kindly treatment of the medical public.

Our pages are no place for theological discussion. Aware that it will be difficult to give even a synopsis of the doctrines of the book without intruding upon the difficult grounds of biblical exegesis, we shall nevertheless examine it purely with reference to its anatomical and physiological bearings, leaving the great questions of theology and politics which hang upon it, as nearly undisturbed as possible.

We subjoin a brief abstract of the doctrines or propositions asserted in "Types of Mankind," and shall briefly examine the proofs on which they rest.

1. All countries are found peopled by peculiar races, associated with a peculiar flora and fauna.

2. These differences of races are so remarkable that they are easily recognizable both in the living man, and in monumental and fossil remains.

3. These differences have existed unchanged for a period of more than five thousand years.

4. The blending of these several races is productive of hybridity to a certain extent, dependent on the proximate, or diverse, characters of the races conjoining.

5. Climatic influences do not change or modify the peculiar character of a race.

The first proposition is the same as that propounded by Agassiz in his lecture on the unity of races, reported in this Journal a few months since. The same ideas, accompanied by an illustrative chart, are embodied in his contribution to "Types of Mankind."

In it he asserts, distinctly, the great principles which govern the book, and supports them by his geographical division of the earth into zoological provinces. That this division really exists in the present order of things there can be no doubt. The various races of men are found in sections of the world peculiar to themselves, and surrounded by a Flora and Fauna of their own. Thus, the arctic regions has the Esquimaux, the polar bear, the seal, the eider duck, and the lichen, as its distinctive types; found in no other regions, having an organization peculiarly adapted to that in which they exist, and which unfits them for existence in the other zones. In the temperate zone we have various regions, occupied by races peculiar to them, and which, though prevented by no outward circumstances from emigration, still cling to their native soil; their history dating back to beyond the historical era; but proved by monumental and fossil remains to have existed unchanged in type from the remotest ages. In the oldest tombs of Egypt the distinctive features of the Shemitic, Nilotic and Abyssinian physiognomies, are found as clearly marked as at the present day. So, too, the animal tribes are equally unchanged in type, and from these and other circumstances, such as the aboriginal races of America, it is argued that they are autocthynes — the primordial inhabitants of their several regions. It is further asserted that this immobility of type is an evidence of adaptation to the features of soil and climate, that if we find that so little change has occurred in a period which dates back to the Diluvian era, or beyond it, we must thence conclude that these races have not only a longer existence than has been ascribed, but that they spring from diverse origins or creations; that when the divine architect first peopled the earth, he placed upon it, not a single pair, but created them by nations, and set upon them those marks by which they may yet be recognized.

The Mosaic record implies that this was the case in the creation of animals. No allusion is found in the first chapter of Genesis to single pairs of animals; "Male and female created he them," is the language of the text; and no reference is given to the number of males or females, or to the locality of their creation.

The second proposition must also be conceded. We believe that most observant people would readily distinguish a Jew, a Mongol, or a Celt from any of the Germanic races. Certainly the wide difference that exists between a Bushman and an European is sufficiently clear. If, also, we are right in supposing that the engraving of the monumental paintings of Egyptian tombs are faithful fac-similes, then we must concede that the various races there represented as Nilotic, Shemitic, Malay, Pelasgian, Nubian, and negro, were as clearly recognizable then as now. Those in the habit of observing crania can also very readily detect in their conformation the race to which they belong.

The third statement is based upon recent Egyptian researches, and involves, in a great measure, the whole question at issue. If these differences are traceable to a period ante-dating the Mosaic flood, or even one succeeding it a few centuries, we are left in the dilemma of supposing that an unrecorded miracle must have intervened after the departure of Noah from the ark, and before the journey of Abraham to Egypt, three centuries later.

The oldest histories of the human race are the Bible, and the monumental records of Egypt. Added to reasonings from these, we must also look to those fossil remains which exist in tombs in the old world, or in the aboriginal mounds of America. Added to this, we may search in modern history for such evidences of the mutability of types as may be offered in the history of those nomadic races which are said to have changed their complexion by emigration, and consequent amalgamation.

Prichard, in his splendid "Natural History of Man," adduces a multitude of evidences of mutability, both in animals and man. To some of these we shall allude under the head of hybridity — the question now is, can we trace back human types in their present condition to a period of say five thousand years ago? If we find that those types then existed, which are now recognized, we may conclude that they must possess a permanency, which, while it does not settle the question of unity, renders it evident that they must have lived for a period of great, but indefinite length, preceding the five thousand years.

The Jewish race, as pictured on the Egyptian monuments, is the same in physiognomy and cranial conformation as now. The history of this people is a strong point in the argument against unity. When Abraham, who, according to his genealogy, must have seen and known Noah, and who is said in some of those apocryphal books which are not included in the christian Old Testament, but are reverenced by the Jews, to have been educated by Shem — when Abraham went down to Egypt, three hundred years after the flood, he found there a race distinct from his own, advanced in civilization, living in cities, the pyramids already built, and with a history dating back to an indefinite period. So, too, on those monuments, left as the only historical record of a period whereof the memory of man runneth not back to the contrary, are found the Egyptian ideal of the four races of man — red, yellow, white and black. There, too, is seen the prognathous face of the negro, as unmistakable as it now is in our streets; there is the grand continuous line of the Grecian countenance; there, the hooked nose of the Syro-Arabian; and the oval face, aquiline nose, and receding chin of the dominant Nilotic race.

But, fortunately for the length of our paper, it is not necessary to consider this point further. Prichard, the principal authority of orthodox ethnology, and the strongest opponent of modern theories, has conceded that the human race must have existed for "chiliads of centuries."

4. *Hybridity.* — While we reserve for a future occasion the full consideration of this subject, we wish here to state the principal bearing it has on the question of races. We quote from Prichard* the following fair statement of the issue; providing his premises are correct.

"It seems to be the well-established result of inquiries into the various tribes of organized beings, that the perpetuation of hybrids, whether of plants or animals, so as to produce new and intermediate tribes, is impossible.

"Now, unless all these observations are erroneous, or capable of some explanation that has not yet been pointed out, they lead, with the strongest force of analogical reasoning, to the conclusion that a number of different tribes, such as the various races of men, must either be incapable of intermixing their stock, and thus always fated to remain separate from each other; or, if the contrary should be the fact, that all the races to whom the remark applies are proved by it to belong to the same species."

This proposition is sustained by Prichard from the very numerous instances of mixed races with which all Americans are familiar. But, admitting the fact *of admixture, we have still left the problem of the origin of* those differences in color and conformation, which we find in various races.

---

*Natural History of Man. London. H. Baillieu.

Prichard has himself, in a great measure, abandoned the supposition of climatic influence, as a cause of this diversity. The existence of the white, the red, and the black races, in America, under the same sky, but utterly unchanged by its influence, is too strong a fact to be over-ridden by mere theory. Prichard, then, although assigning to climate a degree of influence, chooses to suppose that these changes are effected by accident, that the first black man may have been a monstrosity, perpetuated in succeeding generations. Others choose to suppose a miraculous interference of the Almighty, at some time immediately subsequent to the deluge. Such a miracle should have found a place in Holy Record. If we turn to accidental causes, as suggested by Prichard, we see at once that the instances given are insufficient, and sometimes erroneous. Thus the Black Jews of Hindostan, are by him mentioned as an original Shemitic stock, which has become black during a residence in that tropical climate. But the highest authority among educated Jews, the Rabbi Rafail of New York, as well as the ancient records of the people themselves, prove them to be a black race, converted to Judaism, and living side by side with, but despised by, a more powerful race of white Jews.

Charles Hamilton Smith,* after speaking of the insufficiency of the climatic theory, lessens the number of races to three aboriginal types, and states the historical argument as follows:

" * * * As there are only three who attain this typical standard, we have in them the foundation of that number being exclusively original.

"This inference is further supported by facts, which show, if not a succession of distinct creations of human forms, at least probabilities, that their different characteristics are of a remoter date than the last great cataclysis of the earth's surface; for the admitted chronological data do not give a sufficient period of duration between that event and the oldest picture sculptures of Egypt, to sanction the transition from Caucasian bearded to the negro woolly-haired, or *vice versa*, as both appear on the monuments. In that case, the operation of the decided changes would have passed through all their main gradations in three or four centuries, without any subsequent perceptible addition in as many thousand years; or should the beardless stock, which never becomes intensely black, be regarded as intermediate, the difficulty is increased; and it may be remarked, in addition, that the first admissible appearance of this type, in historical records of the west, is incomparably more recent."

---

*The Natural History of the Human Species. London : Henry G. Bohn.

It is not assuming too much, to say that the non-perpetuity of hybrids is "a well established fact." The experiments of Hellenius, of Stockholm, produced a third generation from a cross between the sheep and the deer; but single instances of this kind should not rest against the great and self-evident law adduced by Prichard. But there are some well-known exceptions to this law, which, though not needed, fortify the position of Morton that there are degrees of hybridity. It is probable that uniform periods of gestation have a large influence on the prolificacy of hybrids. Cuvier assumed that the capability of producing offspring was the true test of hybridity. But hybridity is not a unit. It has its degrees, depending on the proximate, or diverse, characters of races. Thus, the offspring of the Celt, and Pelasgian, are not only prolific *inter se*, but present a blending of the two types; one of them predominating. This hybrid is healthy, and does not impair either race.

But when we look at a more distant union, such as a blending of the European with the negro, we see hybridity manifested by a mental improvement in the negro, with a deterioration in the white, by a loss of the physical vigor of either race, incapacity for severe labor, tendency to scrofulous disease, and finally to a short life in the individual, and an unprecedented mortality, and rapid disappearance of the mongrel race.

We find that we have already given all the space we deem necessary to the fifth proposition, relative to climatic influences. We wish, in conclusion, while acknowledging the high character of this work, the force its of arguments, and their apparently unanswerable nature, to say that he who carefully reads Prichard, will find another side to the question; that, above all, it ill becomes any thoughtful mind to decide hastily on so important a subject. The gradual collection of facts, the earnest labors of those espousing either side, will eventually bring us somewhere near the truth. Of that we need have no fear. In all the workings of the human mind, wander though it may from the faith of religion, it constantly returns to the necessity of a religious belief, and feeling its own weakness and insufficiency, it seeks for rest upon a stronger arm.

This book will meet with an opposition which the metaphysics of Voltaire, and Hume, and Paine, did not deserve. The sentiment so natural to the human breast, that sentiment which seeks for an individual God, known as well through words as works, speaking as to children, supporting sustaining, offering hopes of higher happiness, and of final release from present pain and misery, is stayed up on every side by the habits and education, as well as the hopes and fears, of mankind. From the baptism to the funeral, the rites of religion accompany us, and mould our minds. A thousand things speak daily of this reverence for the Bible, and keep the sentiments constantly employed. The silent spire that points to Heaven; the "dim religious light" of churches; the deep intonations of the organ; the pealing of the bell; the Sabbath day of rest — all are constant agencies, which have grown dear to the hearts of men, and inspire an inborn opposition against any doctrines which attack this plan of revealed religion, which is so consonant to their feelings.

We have thus stated the tendencies of "Types of Mankind," with the view to bring forcibly before the reader the results which an espousal of its theories must accomplish. We do not do it to establish a prejudice against it, but the tenets of the work are so clearly enforced, that the prudent mind should fully estimate the great and radical changes they propose, before it lends to them its adherence.

And it is, perhaps, right that we should here recall that other argument which insists that the Bible is a point above attack; that anything in theory, or alleged fact, which conflicts with it, is necessarily false, and should not be investigated. We are aware that this is a received dogma with many minds of the highest character, and that they accordingly denounce any work or author which approaches this subject.

But if God's works are a legitimate and allowable study, his word will not conflict with them. It is only necessary to be sure that we read those works aright; that we not only fully understand a single fact, but that we recognize the bearings which all other facts must have upon it. In a word, we must know all the facts attainable, and place upon them a just construction.

To the medical profession is, in a great measure, confided the conduct of this discussion. It is our sincere belief, that, further than an honest recognition of them, this subject should be studied without reference to its consequences. The prior understanding of results will be an inducement to careful induction, and to cautious generalization. Three great medical names speak out already in debate. Morton, gifted in all learning, and the highest ornament of anatomical science; Nott and Agassiz, are surely entitled to a hearing from the profession they honor. No body of men is so able to sift their arguments, and to judge rightly on their merits, as those whose anatomical studies have enabled them to recognize the conformation of races, and whose physiological knowledge can solve the problem of comparative mental power, of mutability of types, and of hybridity. H.

